# Unraveling the Enigma of Perforating Itches: A Comprehensive Report of Reactive Perforating Collagenosis in Three Patients

**DOI:** 10.7759/cureus.44531

**Published:** 2023-09-01

**Authors:** Praveen BK, Dinesh Asati, Hemlata Panwar, Deepti Joshi

**Affiliations:** 1 Department of Pathology and Laboratory Medicine, All India Institute of Medical Sciences, Bhopal, Bhopal, IND; 2 Department of Dermatology, All India Institute of Medical Sciences, Bhopal, Bhopal, IND

**Keywords:** abnormal collagen metabolism, perforating dermatosis, diabetes mellitus type 2, umbilicated papules, prurigo nodularis (pn), reactive perforating collagenosis

## Abstract

Reactive perforating collagenosis (RPC) is a rare dermatosis where dermal connective tissue erupts through the epidermis, resulting in diverse clinical manifestations such as umbilicated papules with crusting and excoriated nodules with central puncta. Associated with systemic disorders like diabetes mellitus, chronic kidney diseases, and autoimmune conditions, RPC's pathogenesis involves abnormal collagen metabolism, immune dysfunction, genetic predisposition, and environmental triggers. Histopathological examination reveals vertically oriented shallow cup-shaped invaginations containing degenerated collagen fibers, aiding diagnosis. Treatment includes managing underlying causes and utilizing options like topical corticoids, retinoids, and phototherapy, with a possibility of spontaneous regression and recurrence. This case report highlights the significance of considering RPC in patients with characteristic skin lesions and severe itching, emphasizing early recognition and accurate diagnosis to optimize patient care. Continued research and collaboration are crucial for improving outcomes in individuals affected by RPC.

## Introduction

Reactive perforating collagenosis (RPC) is a rare form of skin disorder that comes under the spectrum of perforating dermatosis which also includes Kyrle disease, perforating folliculitis, and serpiginous perforating elastosis [1]. RPC is characterized by the spontaneous eruption of dermal connective tissue through the epidermis. Usually, RPC is associated with systemic disorders like diabetes mellitus, chronic kidney diseases, and collagen disorders. Other associations of RPC include autoimmune disorders like vasculitis, systemic lupus erythematosus, dermatomyositis, and Mikulicz disease [2,3]. A few of the reported cases are also seen in the sites of previous trauma, mosquito bites, and scratchings. A rare form of inherited RPC is seen in infancy or early childhood [3,4].

The usual clinical presentation of RPC is umbilicated, hyperkeratotic, millimetric, or large papules or nodules, with the presence of extensive crust formation. Skin biopsy and histopathological examination are essential for diagnosis [5,6]. This case report focuses on three distinct cases that were observed, highlighting the diverse manifestations of RPC. The observed symptoms ranged from the presence of umbilicated papules accompanied by crusting to the occurrence of excoriated nodules exhibiting central puncta. These cases serve as a significant reminder to healthcare professionals about the crucial need to consider RPC as part of the differential diagnoses for patients who present with characteristic skin lesions accompanied by severe itching. The differential diagnoses includes arthropod bites, dermatofibroma, Elastosis perforans serpiginosum, Kylre disease, multiple keratoacanthoma, perforating folliculitis, and prurigo nodularis. This comprehensive case report has highlighted the clinical presentations, histopathological findings, and potential underlying factors associated with RPC.

## Case presentation

This report describing three distinct cases observes the diverse manifestations of RPC and thus underscores the importance of considering RPC in the differential diagnosis of patients presenting with characteristic skin lesions and severe itching.

Case 1

A 66-year-old elderly woman presented with complaints of multiple umbilicated papules or nodules with center crusting present over bilateral arms, lower limbs, and lower back. The skin lesions were associated with severe itching and serous discharge. The patient was a known case of chronic kidney disease and diabetes mellitus on irregular oral medications. The clinical differential diagnosis was prurigo nodularis, RPC, and perforating folliculitis.

**Figure 1:**
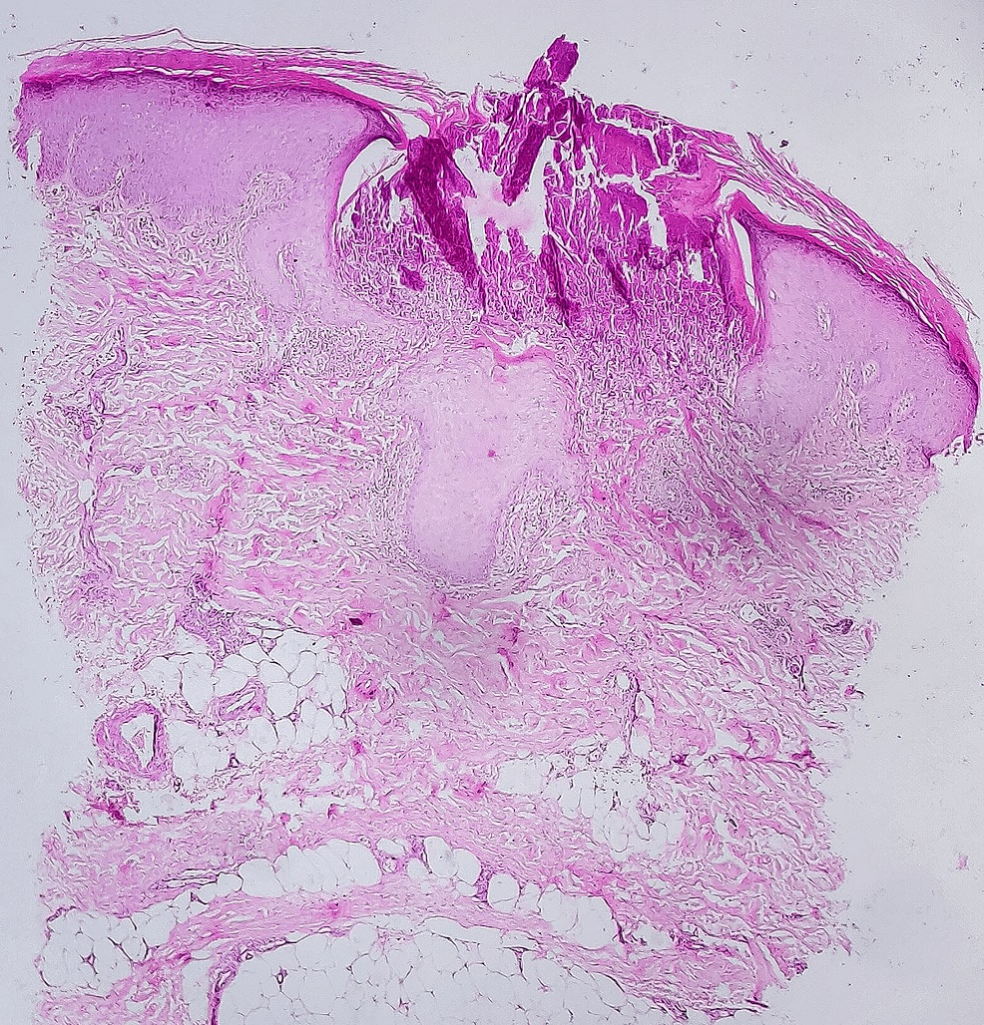
Case 1, HPE section revealing vertically oriented and shallow cup-shaped invaginations within the epidermis, creating short channels filled with densely packed degenerated collagen (H&E stain, 4x). H&E: hematoxylin and eosin; HPE: histopathological examination

**Figure 2:**
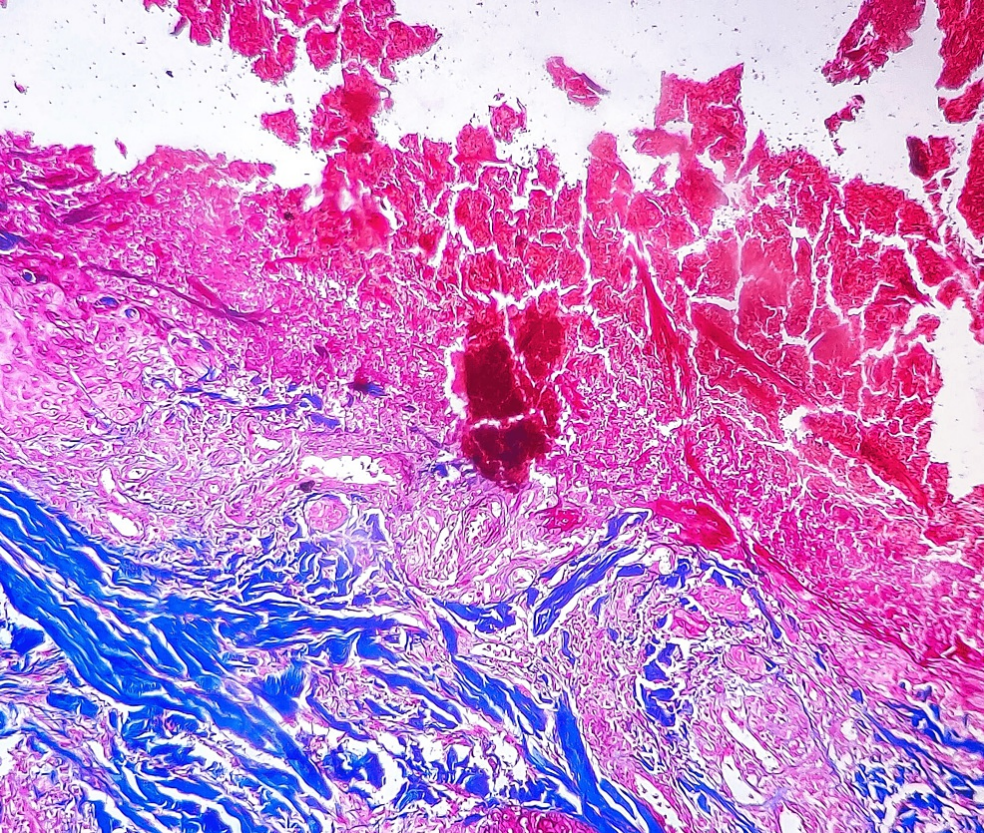
Case 1, MT stain revealing blue-stained collagen fibers within the epidermal channels. The invaginations remain vertically oriented and shallow, and the collagen fibers show signs of degeneration and dense packing (MT stain, 20x) MT: Masson's trichrome stain

Histopathological examination showed vertically oriented shallow cup-shaped invagination within the epidermis forming a short channel with densely packed degenerated collagen (Figure 1). Masson’s trichrome stain (MT) highlighted blue-stained collagen fibers perforating the channel and extending to the surface (Figure 2).

Case 2

**Figure 3:**
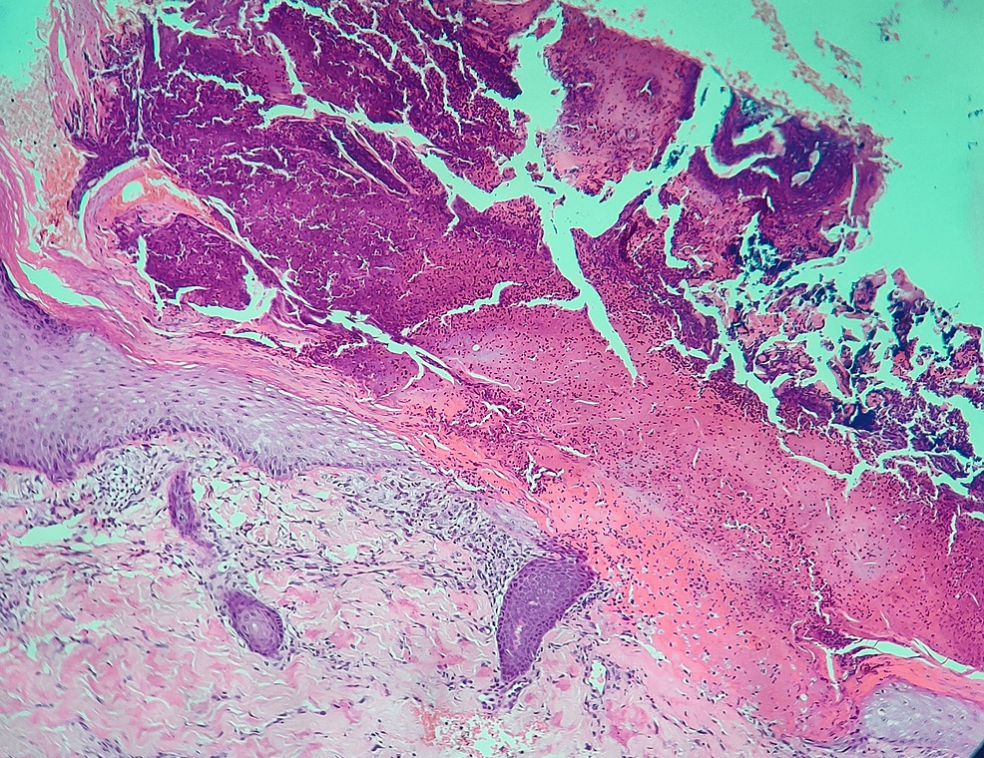
Case 2, HPE section showing the presence of a plug containing basophilic to eosinophilic degenerated material and parakeratotic debris within the epidermis (H&E stain, 4x) H&E: hematoxylin and eosin; HPE: histopathological examination.

**Figure 4:**
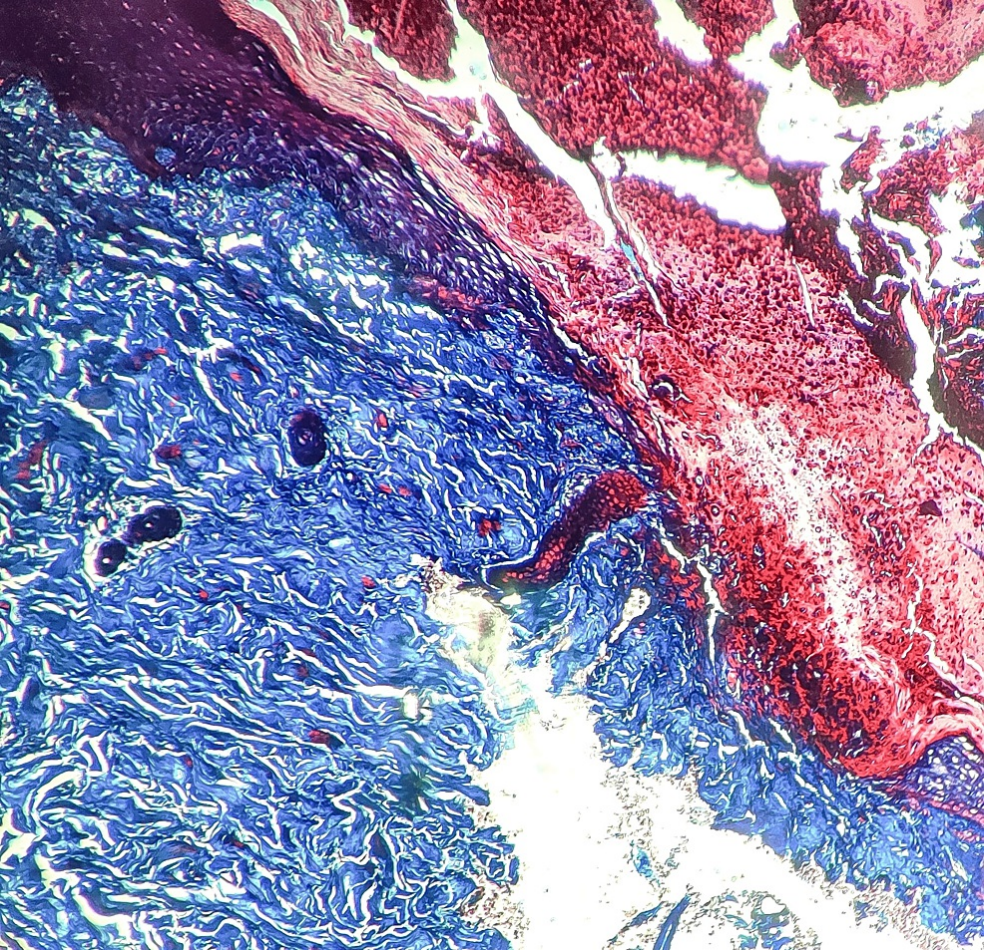
Case 2, MT stain highlighting degenerated collagen fibers in the upper papillary dermis (MT stain, 4x) MT: Masson's trichrome stain

A 33-year-old woman presented with complaints of multiple excoriated papules, a few showing central puncta over bilateral lower limbs and lower back. The lesions were associated with severe itching. The patient had a history of gestational diabetes mellitus and was on ayurvedic medications for the past three years. The current glycated hemoglobin (HbA1c) level was raised (7.5%). The differential diagnosis considered for this case was RPC and prurigo nodularis. Histopathological examination showed epidermis with a plug containing basophilic degenerated material and parakeratotic debris (Figure 3). The upper papillary dermis shows degenerated collagen fibers highlighted by MT (Figure 4).

Case 3

**Figure 5:**
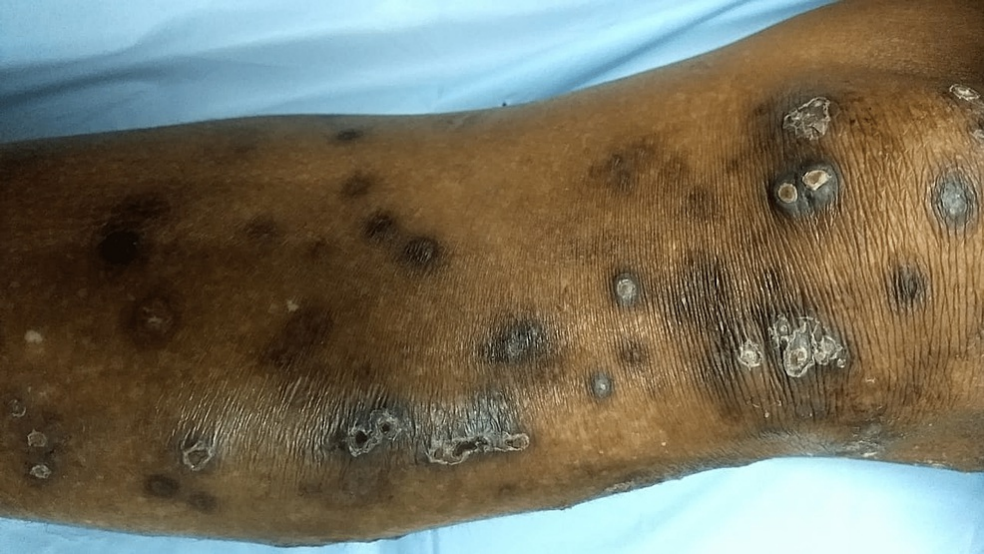
Case 3, Clinical image revealing cluster of multiple small hyperpigmented flat-topped papules with central punctum over the knee region. These papules exhibit a hard-to-firm texture and the central punctum represents the epidermal elimination site

A 19-year-old woman presented with complaints of multiple small hyperpigmented flat-topped, hard-to-firm papules with central punctum (Figure 5). The lesions were associated with severe itching. The patient had a past history of trauma (a fall from a bicycle) on the same site 10 years prior to presentation. This patient’s brother had also similar lesions and took some traditional herbal treatment for the same.

**Figure 6:**
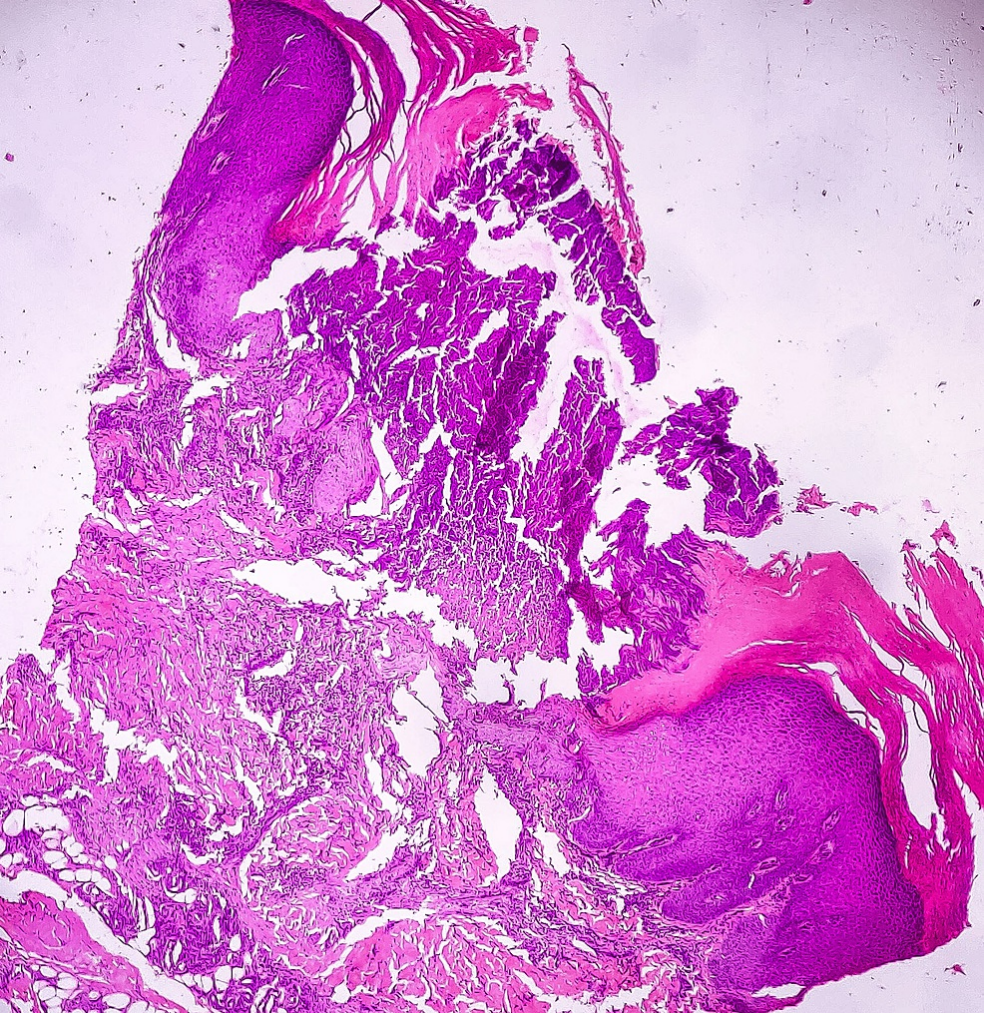
Case 3, HPE section shows canalization of degenerated collagen bundles and vertically oriented collagen fibers. The affected area shows an overlying parakeratotic plug, accompanied by blunting of the rete ridges (H&E stain, 4x). HPE: histopathological examination; H&E: hematoxylin and eosin stain.

**Figure 7:**
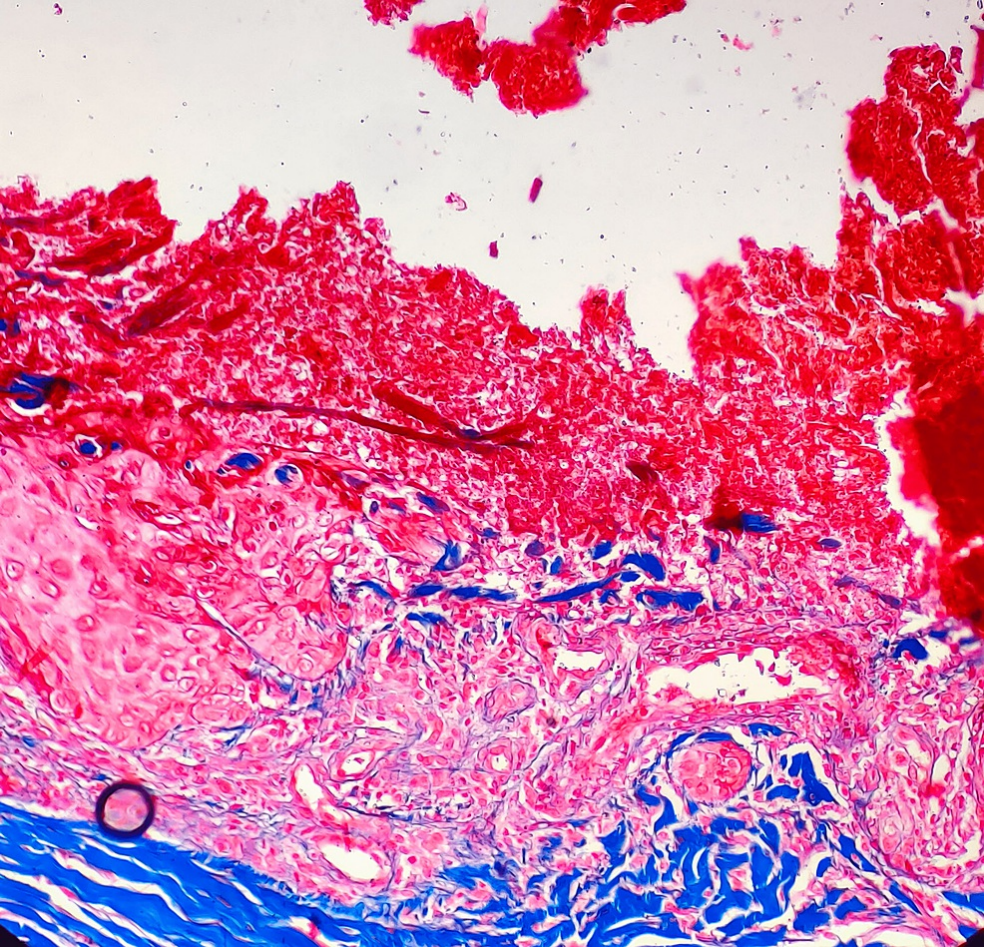
Case 3, MT stain highlighting degenerated type of collagen within the perforating channel (MT stain, 20x) MT: Masson's trichrome stain

Histopathological examination of skin biopsy showed canalization of degenerated collagen bundles and vertically arranged collagen with overlying parakeratotic plug with blunting of rete ridges (Figure 6). MT staining showed degenerated type of collagen fibers within the perforating channel (Figure 7).

Management

All three cases were managed using a combination therapy approach, consisting of systemic retinoid therapy with acitretin, along with the local application of steroids. Additionally, the primary treatment course involved addressing the underlying long-standing diabetes through insulin therapy. Encouragingly, the first two patients exhibited significant improvement and experienced notable relief from their symptoms. Unfortunately, the third patient was lost to follow-up, making it difficult to assess the response to the treatment in their case.

## Discussion

RPC is one of the most common forms of perforating dermatosis. There are two types of RPC reported so far, the first and least common one being the hereditary form, usually seen in infancy/early childhood while the second and most common is the acquired form, seen in patients with diabetes mellitus and renal failure [7,8]. The familial form, considered to have an autosomal recessive form of inheritance, is common in males while the acquired form occurs more commonly in females. RPC usually affects the limbs and extremities [9].

The pathogenesis of RPC remains multifactorial and not fully understood. Several factors have been suggested to play a role in the development of RPC. Firstly, there is evidence indicating that abnormal collagen metabolism may contribute to the condition, with alterations in collagen fiber production and breakdown [10]. Secondly, RPC has been associated with immune dysfunction, as observed in various autoimmune and inflammatory conditions like rheumatoid arthritis and lupus. It is believed that an abnormal immune response in the skin could trigger the formation of papular lesions in RPC. Thirdly, while RPC is not considered hereditary, some studies have proposed a genetic predisposition to the disease, where certain genetic variations may increase the risk of developing RPC [11]. Lastly, environmental triggers may also play a part, as factors such as sunlight exposure or specific chemicals could potentially initiate or worsen RPC symptoms [10].

Overall, the pathogenesis of RPC is complex and multifactorial. More research is needed to fully understand the underlying mechanisms of the disease and to develop effective treatments. Superficial trauma can lead to necrobiosis of dermal collagen in susceptible individuals. This damaged collagen can later undergo transepithelial elimination through the damaged epidermis. This must be the associated causative factor in Case 3. Long-term hyperglycemia can also cause altered collagen I and II [11]. A few hypotheses were also regarding the relationship between diabetic vasculopathy and nephropathy and RPC. However, that does not explain the RPC in nondiabetic patients [12]. It is, in rare cases, also associated with solid tumors, AIDS, lymphomas, Hodgkin's disease, systemic lupus erythematosus, and dermatomyositis, and during treatment with tyrosine kinase receptor inhibitors like erlotinib [7].

The clinical features include umbilicated, millimetric, hyperkeratotic, larger papules or nodules, with excoriations and crust formation. The lesion erupts as a papule and grows wider and deeper over one month, then a central crater develops with a compact horny plug appearance over the periphery of the lesion. This may regress over one month leaving behind a scar or hypo/hyperpigmented area [6,9]. This is associated with pruritis of varying degrees. The role of scratching secondary to pruritus and repeated micro-traumatisms is a triggering factor.

RPC is primarily diagnosed through biopsy and histopathological examination. Microscopically, RPC is characterized by the presence of vertically oriented shallow cup-shaped invaginations within the epidermis, forming short channels that extend from the epidermis into the underlying dermis [4]. These channels contain densely packed degenerated collagen fibers, which can be highlighted using special stains such as MT. MT stain imparts a blue color to collagen fibers, aiding in the visualization and identification of the degenerated collagen fibers in RPC. Within some of these channels or invaginations, a plug composed of basophilic degenerated material and parakeratotic debris may be observed as noticed in Case 2. Histopathological examination may also reveal the blunting of rete ridges, which are the epidermal extensions that project into the dermis. By analyzing these microscopical features, along with the utilization of special stains like MT stain, a definitive diagnosis of RPC can be established [2,4,11,12].

Treatment of RPC involves prompt and aggressive treatment of the underlying cause. Specific therapeutic options for RPC include topical corticoids, local and systemic retinoids, and UVB-TL01 phototherapy but there are no clear guidelines for treatment. Excimer laser therapy is also being advised but is under scrutiny [13].

Treatment often may result in symptomatic results but may result in recurrence [13]. It is also noted that without treatment, familial RPC often regresses spontaneously in approximately 10 weeks. Recurrence is more frequent in familial cases [12,14].

## Conclusions

RPC is a rare and intriguing skin disorder characterized by the eruption of dermal connective tissue through the epidermis. This comprehensive case report has highlighted the clinical presentations, histopathological findings, and potential underlying factors associated with RPC. By enhancing our understanding of RPC and emphasizing the importance of early recognition, accurate diagnosis, and multidisciplinary approaches, we can optimize patient care. Continued research and collaboration are essential in furthering our knowledge and improving outcomes for individuals affected by RPC.
